# Giant benign intrathoracic schwannoma: a decade-long progression towards fatality

**DOI:** 10.1186/s13019-023-02375-2

**Published:** 2023-11-14

**Authors:** Michiyo Miyawaki, Takashi Karashima, Miyuki Abe, Yohei Takumi, Takafumi Hashimoto, Ryotaro Kamohara, Atsushi Osoegawa, Kenji Sugio

**Affiliations:** 1 Department of Thoracic and Breast Surgery, Oita University Faculty of Medicine, 1-1 Idaigaoka Hasama-machi Yufu, Oita, 879-5593 Japan; 2 Department of Thoracic Surgery, Oita Prefectural Hospital, 8-1 Bunyo 2-chome, Oita, 870-8511 Japan

**Keywords:** Chest wall tumor, Schwannoma, Giant tumor, Life-threatening

## Abstract

**Background:**

Intrathoracic neurogenic tumors arise from sympathetic nerve trunks and intercostal nerves; more than 90% are benign. Schwannomas are the most common histological variety, but fatalities due to giant schwannomas are rare.

**Case presentation:**

We report a case of a 65-year-old woman who presented with chest pain and cough. Computed tomography (CT) revealed a large left chest wall mass of 130-mm in size, and the patient was referred to our department. Tumor biopsy was performed under local anesthesia, and a diagnosis of schwannoma was made. Ten years previously, a 30-mm tumor had been noted in the left third intercostal space by a previous doctor, but follow-up had been interrupted owing to depressive disorder. Although we planned to perform intercostal artery embolization followed by chest wall tumor resection, the patient did not consent to surgery due to uncontrolled depression. After four months, she developed respiratory failure caused by compression due to an enlarged tumor and died. Autopsy also revealed a benign schwannoma with no malignant findings.

**Conclusions:**

Although schwannomas are benign tumors, there are some very rare cases in which they can become huge and life-threatening. Therefore, a benign tumor should not be neglected, and if surgery is not possible at the time of diagnosis, a regular follow up is necessary, in order not to miss the right timing for surgery.

## Background

**Fig. 1:**
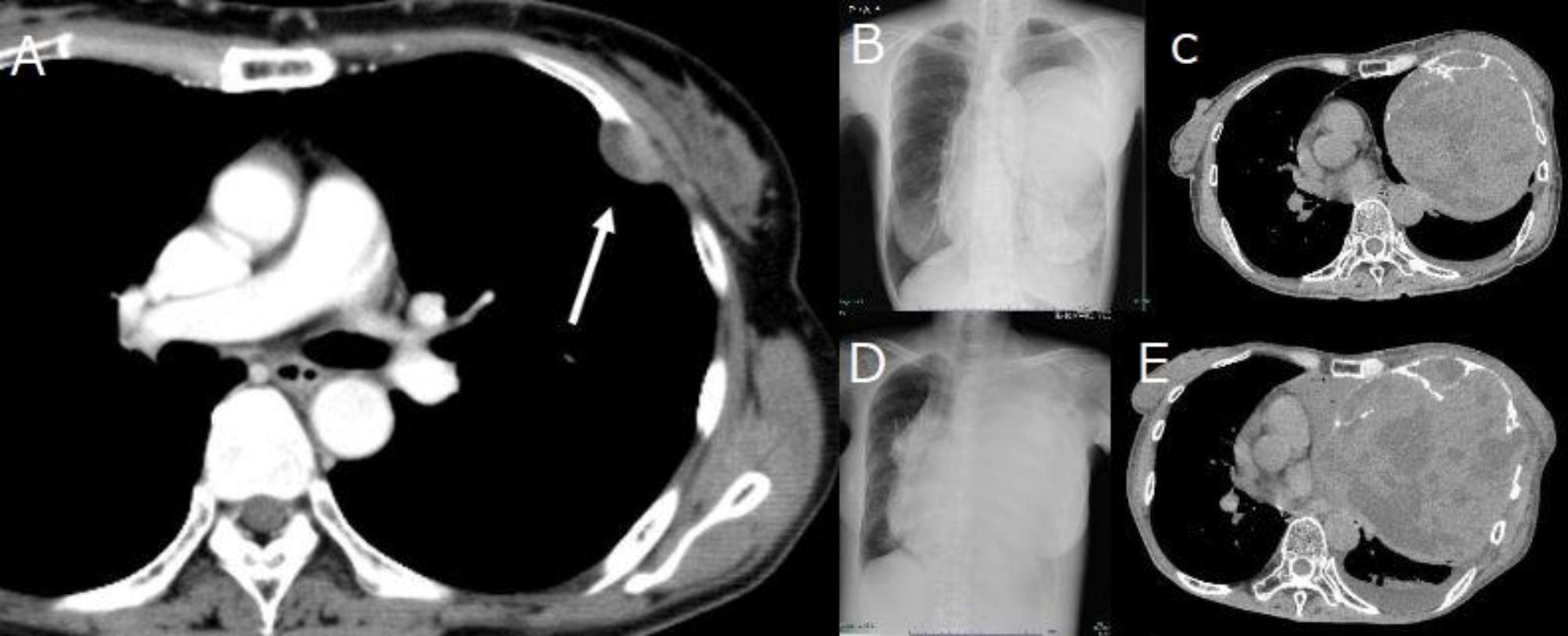
**(A)** A CT taken 10 years previously shows a small nodule (3.0 × 1.5 cm) in the left third intercostal space(arrow). A chest radiograph **(B)** and CT scan **(C)** at the time of referral to our hospital, show a large left chest wall mass of 130-mm in size. Tumor has a fluid component and calcification inside. The ribs are also partially destroyed. A chest radiograph **(D)** and CT scan **(E)** show further tumor enlargement, a completely atelectatic left lung, and compression of the mediastinum

**Fig. 2:**
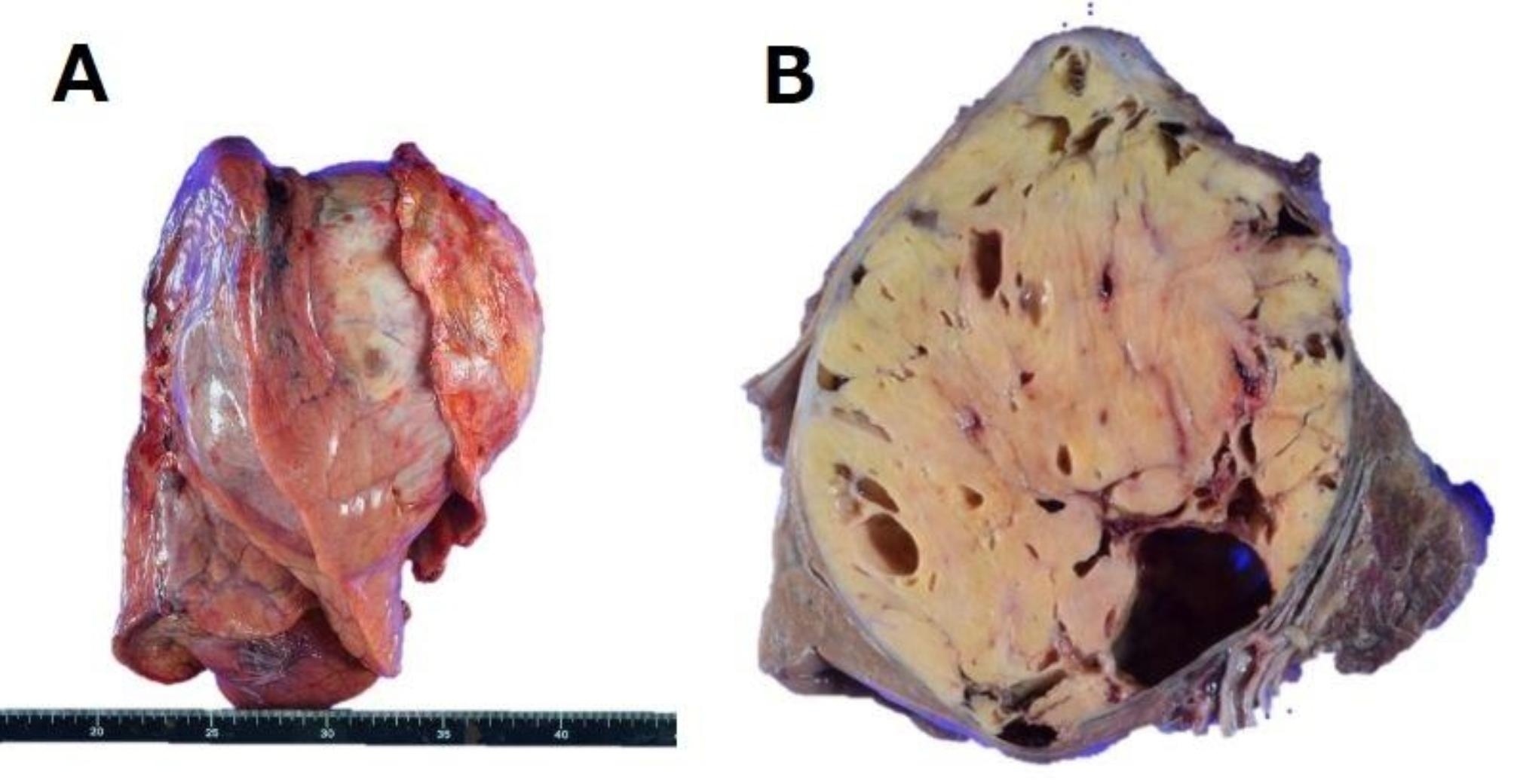
A specimen of the tumor removed at autopsy. The tumor was 15 cm in size and was a single mass fused with the left lung and chest wall **(A)**. Findings on the segmental surface after fixation **(B)**. The tumor had partially cystic formation, involving and destroying the ribs

**Fig. 3:**
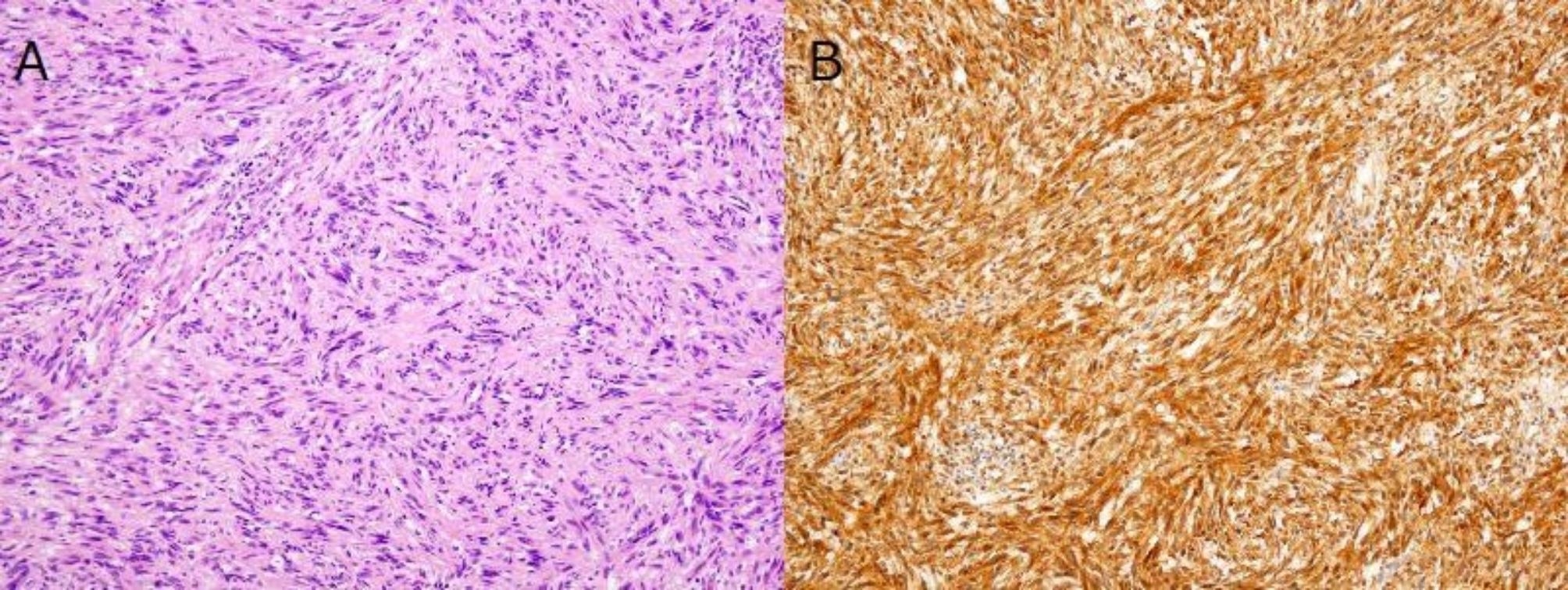
Hematoxyline and eosin staining showed **(A)**, that the tumor was composed of cells with fewer atypically spindle-shaped nuclei, arranged in bundles. There was no nuclear mitosis, and the tumor was predominantly cell-dense Antoni A ( Magnification×100). Tumor cells showed strong positivity on immunohistochemistry for S100 protein **(B)** (Magnification×100)

Neurogenic tumors of the chest that arise from sympathetic trunks and intercostal nerves are often located in the posterior mediastinum. Neurogenic tumors in the adult are commonly of nerve sheath origin and 98% are benign. More than 95% of these are either schwannomas or neurofibromas [1]. Neuromatosis type 1 (NF1) von Recklinghausen disease is characterized by multiple neurofibromas that develop malignantly in 1–13% of cases. However, sporadic schwannomas usually do not become malignant, and the lethal growth of schwannomas of intercostal nerve origin is extremely rare. Here we present a case of a giant benign intrathoracic schwannoma: progressive enlargement over a decade resulting in fatal outcome. 65-year-old woman was referred from a local hospital for further examination of a giant chest wall tumor. Ten years previously, at that hospital, a 30 mm chest wall tumor had been noted in the left third intercostal space (Fig. 1A), but she did not receive any follow-up due to severe depression. She visited the hospital after 10 years with a chief complaint of productive cough and chest pain, and a large left chest wall mass 130-mm in size was identified on chest radiograph and computed tomography (CT) and X-ray examinations (Fig. 1B, C). The tumor was compressing the left pulmonary hilum. She was 159 cm tall, weighed 38 kg, and experienced emaciation and prostration. Blood counts and coagulation function test findings were within normal limits. Under local anesthesia, incisional biopsy of the tumor revealed a schwannoma. One month after biopsy, the left lung was completely atelectatic. A respiratory function test indicated a marked restrictive disorder pattern: %vital capacity (%VC) of 53.3% and forced expiratory volume 1.0 (sec) % (FEV 1%) of 71.3%. We planned to perform tumor resection involving the 2nd, 3rd, and 4th ribs, and chest wall reconstruction, following intercostal artery embolization performed the day before. However, the plan was canceled due to a worsening of her depression. Four months later, the patient was transported to the emergency room with progressive dyspnea and severe back pain. Although surgery was reconsidered, left lung congestion, right lung pneumonia, and hypotrophy had progressed (Fig. 1.D, E). The patient died of respiratory failure without life-prolonging measures. This was 225 days after her first visit to our clinic. As the family provided consent for autopsy, a pathological autopsy was performed (Fig. 2). The pathological diagnosis was an Antoni type A schwannoma of the chest wall (Fig. 3). No malignancy was observed in the tumor. In addition, atelectasis of the left upper lobe and diffuse alveolar damage to the right lung were observed. Autopsy revealed that the cause of death was diagnosed as respiratory failure due to pneumonia and pulmonary edema, which occurred as a result of decreased reserve capacity of respiration and circulation caused by compression of tumor, and other causes such as myocardial infarction and pulmonary embolism were ruled out.

## Discussion and conclusion

Schwannomas are typically solitary lesions in deep or superficial soft tissues, usually occurring along the nerves of the head, neck, and upper and lower extremities. In the thoracic cavity, schwannomas are most frequently located in the posterior section of the mediastinum, arising from the sympathetic trunk or intercostal nerves. In soft tissue tumors, with the exception of lipomas, hemangiomas, and desmoid tumors, tumors larger than 5 cm are more likely to be malignant. Usually, schwannomas are small masses (< 5–6 cm), but several intrathoracic giant schwannomas have been reported. [2–8]. The specific mechanisms associated with enlargement of Schwannoma are not yet fully elucidated, but mutations in the NF2 gene and abnormalities in signaling pathways have been suggested [9]. When the tumor becomes large, it causes symptoms due to pressure on the surrounding tissues, but, no reports of cases of tumor-related death associated with benign intrathoracic schwannoma. This case appears to be a rare and valuable instance where a benign tumor inexplicably grew significantly over a period of 10 years, leading to death due to reasons such as missed surgical timing resulting from the lack of follow-up and the presence of comorbid psychiatric illness.

Resection of giant tumors is challenging because of their enormous size and serious adhesion to surrounding organs. It may be necessary to perform a combined resection of adherent tissue. The main challenge is how to control the amount of bleeding. When the tumor is huge and a poor surgical exposure is presupposed then, piecemeal removal is the only choice because an en-block resection is impossible. One strategy to reduce bleeding is preoperative embolization [10], which we also planned to do this case.

There is currently no effective drug therapy for schwannomas. Neurofibromatosis type 2 (NF2) involves multiple schwannomas and meningiomas in the cranial and spinal nerves. Acoustic schwannomas can grow to a critical size, compress the brainstem, and may lead to death. Resection is not always easy; therefore, several therapeutic agents have been considered. Sagers et al. reported that the combination of an mTOR inhibitor and dasatinib may be effective for the treatment of vestibular schwannomas [11]. In addition, a report on the efficacy of the vascular endothelial growth factor (VEGF) receptor vaccine for NF2 [12] and a multicenter, double-blind, randomized, controlled trial investigating the efficacy and safety of bevacizumab are ongoing.

In conclusion, generally, schwannomas are benign and asymptomatic; we therefore sometimes do not pay much attention to them. However, in some cases, tumors gradually become larger and seriously compress the neighboring mediastinal organs, leading to death.

Therefore, a benign tumor should not be neglected, and if surgery is not possible at the time of diagnosis, a regular follow up is necessary, in order not to miss the right timing for surgery.

## Data Availability

All data and materials are available upon reasonable request from the cor‑responding author.
